# Gastric intestinal metaplasia regression in United States population: A retrospective longitudinal study

**DOI:** 10.1002/jgh3.70005

**Published:** 2024-08-19

**Authors:** Akram I Ahmed, Ahmed El Sabagh, Claire Caplan, Arielle Lee, Won K Cho

**Affiliations:** ^1^ Gastroenterology Department Cleveland Clinic Florida, Digestive Disease Institute Weston Florida USA; ^2^ Department of Medicine MedStar Washington Hospital Center Washington DC USA; ^3^ Georgetown University School of Medicine Washington DC USA; ^4^ Division of Gastroenterology/Hepatology INOVA Health System Leesburg Leesburg Virginia USA

**Keywords:** African American, gastric cancer, gastric intestinal metaplasia, gastric mapping, gastric intestinal metaplasia regression, peptic ulcer disease

## Abstract

**Background and Aim:**

Gastric cancer is a health concern and contributes to cancer‐related deaths. Gastric intestinal metaplasia (GIM) is a premalignant lesion of gastric cancer. Currently, factors associated with GIM regression are under‐investigated. This study aims to assess the rate of GIM regression and identify factors associated with it.

**Methods:**

This study was conducted at Medstar Washington Hospital Center. We included patients who had GIM between January 2015 and December 2020. Population was divided into GIM persistence or regression. Data included demographics, esophagogastroduodenoscopy findings, *Helicobacter pylori* status, and laboratory results. Statistical analyses included Kaplan–Meier and Cox proportional models to explore predictors of GIM regression.

**Results:**

Among 2375 patients, 9.1% had GIM. Notably, 85 patients had GIM regression and 132 patients had persistent GIM. African Americans constituted (75%) of the regression group and (76%) of the persistence group. Peptic ulcer disease (PUD) was noted in 12.9% of the regression group at baseline, and 5.9% at follow‐up; the persistence group showed 11.4% at baseline and 5.3% at follow‐up (*P* = 0.89). Regression analysis revealed that the presence of PUD was associated with a higher rate of regression (hazard ratio [HR] 2.46, *P* = 0.013). Smoking status showed lower rates of regression (HR 0.54 and 0.62, *P* = 0.038 and 0.169). On gastric mapping, African Americans, Hispanics, and individuals of other races/ethnicities displayed lower rates of GIM regression (HR 0.68, 0.78 and 0.69).

**Conclusion:**

PUD was associated with a higher rate of GIM regression, while smoking showed lower regression rates. Results provide insights into factors influencing GIM regression in African American population and may inform future surveillance and treatment strategies.

## Graphical abstract

Our retrospective study of 217 patients at Medstar Washington Hospital Center found that 39.1% experienced gastric intestinal metaplasia (GIM) regression. Peptic ulcer disease (PUD) was associated with higher regression rates, while smoking showed lower rates. African Americans and Hispanics exhibited lower regression rates compared to Caucasians. These findings highlight the importance of considering demographic and clinical factors in GIM management and surveillance.

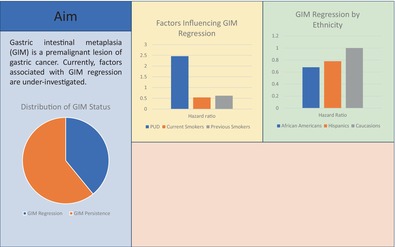

## Introduction

Gastric cancer is the fifth most diagnosed malignancy worldwide.^1^ In the United States (US) population, it accounts for 1.8% (11090) of all cancer deaths annually and has a 5‐year survival rate of 42.9%.^2^, ^3^ Given the low survival rate with a late stage diagnosis, it is important to understand the risk factors associated with gastric cancer to help stratify the cancer risk. In regions with a high incidence of gastric cancer, screening programs led to a reduction in gastric cancer‐associated mortality.^4^, ^5^ In the United States, the incidence of gastric cancer remains low.^2^ Therefore, population‐based screening programs are not economically feasible, and further investigation is needed to identify patients at risk to guide screening for gastric cancer.

Gastric intestinal metaplasia (GIM) is a major pathological precursor for developing gastric cancer. Chronic inflammation causes loss of gastric glands then replacement of gastric mucosa with GIM formation.^6^, ^7^ In addition to atrophic gastritis and dysplasia, GIM is considered an important gastric premalignant lesion.^8^, ^9^, ^10^ GIM is associated with multiple risk factors, such as old age, *Helicobacter pylori* infection, and certain ethnicities.^11^ The role of *H. pylori* chronic infection in the development of gastric malignancy is well established; on the other hand, the effect of *H. pylori* infection on gastric premalignant lesions is more controversial.^7^, ^12^, ^13^ Ethnicity can affect the development of GIM; in the United States, Hispanics have the highest prevalence of GIM, followed by African Americans, and lastly non‐Hispanic Caucasians. Overall, there is wide variation in the reporting of GIM prevalence across large databases because diagnosis involves upper endoscopy and histological evaluation.^14^, ^15^

GIM is an important gastric premalignant lesion in the Correa cascade, which was thought to be an irreversible step in gastric cancer carcinogenesis. However, some recent literature suggests a regression of GIM in some patients,^16^, ^17^ but the reversal of GIM and factors associated with this process remain under‐investigated in the US population, especially in African American population. Thus, the present study is a retrospective longitudinal study undertaken to examine GIM regression in pre‐dominantly African American population to help determine important factors associated with GIM regression thus potentially help develop a surveillance and treatment strategies.

## Methods

### Study design

This is a retrospective, longitudinal study conducted at Medstar Washington Hospital Center, a tertiary hospital in Washington, DC. MedStar Health Institutional Review Board reviewed and approved this study and granted a waiver of consent.

### Study population

The study population was selected from the Pathology Department database. Patients were included if they had an esophagogastroduodenoscopy (EGD) with gastric biopsy showing GIM, with at least one more subsequent EGD with gastric biopsies between January 2015 and December 2020. Patients with GIM were followed longitudinally until the GIM resolved or until the last available EGD. Patients <18 years old or pregnant patients were excluded from the study. The study population was divided into two groups: GIM persistence and GIM regression, based on the result of gastric biopsy on the most recently available EGD.

### Data collection

The electronic medical records of the study patients were reviewed for patient demographics, including age, weight, height, smoking status, and race/ethnicity at baseline. Patient's EGD findings such as *H. pylori* status, pathology report findings, medication use, and laboratory results were reviewed at baseline and follow‐up encounters. In this study, *H. pylori* status was based on biopsy testing. Results of gastric mapping using the updated Sydney system biopsy protocol were collected when available. GIM regression or persistence was determined based on the presence or absence of GIM reported in the follow‐up pathology report. If GIM were present in either the antrum or corpus during follow‐up, it was considered GIM persistence. Regression was defined as the complete resolution of GIM from the stomach.

### Data analysis

We used the D'Agostino‐Pearson test to test normality. Chi‐square with Yate's correction or Kruskal–Wallis rank‐sum was performed to compare the difference between the groups. Kaplan–Meier estimators were calculated, and the curves were plotted to show the probability of GIM Regression at a respective time interval after the baseline. To investigate the effect of predictors on GIM regression over time, we performed the univariate and multivariate Cox proportional hazards regression models. All unadjusted and adjusted hazard ratios with 95% confidence intervals were presented, along with the unadjusted *P* values. We used frequency with percentage for categorical variables to present the data and median with first and third quartile (IQR) for non‐normal continuous variables. Statistical significance was set at a *P* value less than 0.05. All statistical analyses were conducted with R software.

## Results

A cohort of 2375 patients underwent EGD with gastric biopsy from both gastric antrum and body as per our institutional policy‐within the timeframe spanning 2015 to 2020. Among these individuals, 217 patients (9.1%) had a diagnosis of GIM on their gastric biopsy, subsequently undergoing at least one follow‐up EGD. During the follow‐up period, a noteworthy subset of 85 patients (39.1%) had GIM regression (the GIM regression group) over a median follow‐up duration of 413.0 days (interquartile range [IQR] 144–1111). This was juxtaposed with a median follow‐up duration of 547.5 days (IQR 244–1302) for the GIM persistence group, where GIM regression was not observed (*P* = 0.074).

The average age within the study cohort was 62.2 years, with females comprising 52.1% of the total population. Notably, there were no statistically significant differences in age or sex distribution between the two study groups. Further delineation of the demographic characteristics reveals that African Americans constituted 75% of the GIM regression group, while Caucasians, Hispanics, and individuals of other races/ethnicities accounted for 6.2%, 10%, and 8.8% respectively. In the GIM persistence group, the corresponding proportions were 76%, 5.4%, 7.2%, and 11.2%, respectively, and these differences were not statistically different (*P* = 0.852). Detailed baseline characteristics and demographic data are presented in Table 1.

**Table 1:** Baseline characteristics and demographic data

	Overall	GIM regression	GIM persistence	*P*
Total number	217	85	132	
Follow‐up days (median [IQR])	488.0 [195.0, 1260.0]	413.0 [144.0, 1111.0]	547.5 [244.8, 1302.0]	0.074
Follow‐up years (median [IQR])	1.3 [0.5, 3.4]	1.1 [0.4, 3.0]	1.5 [0.7, 3.6]	0.074
Age baseline (mean [SD])	62.2 (13.2)	61.9 (15.0)	62.4 (12.0)	0.819
Sex (%)				
Male	104 (47.9)	40 (47.1)	64 (48.5)	0.947
Female	113 (52.1)	45 (52.9)	68 (51.5)	
Ethnicity/race (%)				
Caucasian	12 (5.9)	5 (6.2)	7 (5.6)	0.852
AA	155 (75.6)	60 (75.0)	95 (76.0)	
Hispanic	17 (8.3)	8 (10.0)	9 (7.2)	
Other	21 (10.2)	7 (8.8)	14 (11.2)	
BMI (%)				
<30	119 (63.0)	42 (60.0)	77 (64.7)	0.623
>30	70 (37.0)	28 (40.0)	42 (35.3)	
Smoking (%)				
Never	99 (48.8)	44 (55.7)	55 (44.4)	0.239
Previous	58 (28.6)	18 (22.8)	40 (32.3)	
Current	46 (22.7)	17 (21.5)	29 (23.4)	

BMI, body mass index; GIM, gastric intestinal metaplasia.

As for endoscopic examinations, 12.9% of patients in the GIM regression group had peptic ulcer disease (PUD) at baseline, which decreased to 5.9% at follow‐up. In contrast, the GIM persistence group displayed 11.4% and 5.3% incidence of PUD at baseline and follow‐up, respectively (*P* = 0.89). While gastritis found at baseline EGD biopsies was similar between the two groups, it was observed in 78.8% of patients in the GIM regression group on follow‐up, compared with 84.1% in the GIM persistence group (*P* = 0.42).

The study cohort displayed *H. pylori* infection in 24.9% of patients at baseline EGD and 13.4% on follow‐up EGD. However, there were no significant differences in the rate of *H. pylori* infection between the two study groups. Factors such as aspirin use, proton pump inhibitor (PPI) use, and smoking status also were not statistically different between the two study groups, with comprehensive data presented in Table 2. Regression analysis revealed that the presence of PUD at the time of GIM detection was associated with a higher rate of regression over time (hazard ratio (HR) 2.05 [1.08–3.89], *P* = 0.03). However, the presence of gastritis at baseline EGD did not yield statistically significant differences between the two study groups. Similarly, gastritis on follow‐up EGD biopsies had no significant impact on the rate of GIM regression (HR 0.76 [0.45–1.29], *P* = 0.31). PUD at follow‐up EGD also did not significantly affect the rate of GIM regression (*P* = 0.26). In contrast, current smokers and previous smokers exhibited HRs of 0.55 and 0.63, respectively for GIM regression when compared with never smokers (*P* = 0.03 and 0.11, respectively). Variables such as age, sex, race/ethnicity, and medication use did not attain statistical significance in the univariate Cox proportional hazards regression model (Table 3).

**Table 2:** Cohort clinical characteristics

		Overall	GIM regression	GIM persistence	*P*
Biopsy bottles (%)	1	101 (46.5)	47 (55.3)	54 (40.9)	0.053
	≥2	116 (53.5)	38 (44.7)	78 (59.1)	
*H. pylori* baseline (%)	No	163 (75.1)	67 (78.8)	96 (72.7)	0.394
	Yes	54 (24.9)	18 (21.2)	36 (27.3)	
*H. pylori* follow‐up (%)	No	188 (86.6)	74 (87.1)	114 (86.4)	1
	Yes	29 (13.4)	11 (12.9)	18 (13.6)	
PUD baseline (%)	No	191 (88.0)	74 (87.1)	117 (88.6)	0.892
	Yes	26 (12.0)	11 (12.9)	15 (11.4)	
PUD follow‐up (%)	No	205 (94.5)	80 (94.1)	125 (94.7)	1
	Yes	12 (5.5)	5 (5.9)	7 (5.3)	
Gastritis baseline (%)	No	35 (16.1)	14 (16.5)	21 (15.9)	1
	Yes	182 (83.9)	71 (83.5)	111 (84.1)	
Gastritis follow‐up (%)	No	39 (18.0)	18 (21.2)	21 (15.9)	0.421
	Yes	178 (82.0)	67 (78.8)	111 (84.1)	
Aspirin use (%)	No	153 (70.5)	60 (70.6)	93 (70.5)	1
	Yes	64 (29.5)	25 (29.4)	39 (29.5)	
PPI usage (%)	No	148 (68.2)	52 (61.2)	96 (72.7)	0.102
Blood type (%)	A	24 (31.2)	7 (25.9)	17 (34.0)	0.305
	B	10 (13.0)	6 (22.2)	4 (8.0)	

GIM, gastric intestinal metaplasia; PPI, proton pump inhibitor; PUD, peptic ulcer disease.

**Table 3:** Univariate analysis of factors associated with GIM regression

Predictor	HR (95% CI)	*P* value
Age	1 (0.98, 1.02)	0.828
Female	0.84 (0.54, 1.28)	0.411
Race/ethnicity (ref: Caucasian)		
African American	0.68 (0.27, 1.72)	0.42
Hispanic	0.78 (0.25, 2.41)	0.665
Other	0.69 (0.22, 2.18)	0.527
Obesity	0.93 (0.58, 1.48)	0.749
Smoking (ref: Never)		
Current	0.55 (0.32, 0.97)	**0.038**
Previous	0.63 (0.36, 1.11)	0.112
*H. pylori* (ref: baseline: No, follow‐up: No)		
Baseline: No, follow‐up: Yes	0.94 (0.41, 2.19)	0.893
Baseline: No, follow‐up: Yes	0.86 (0.47, 1.58)	0.633
Baseline: Yes, follow‐up: Yes	0.92 (0.37, 2.29)	0.854
PUD at baseline	2.05 (1.08, 3.89)	**0.027**
PUD at follow‐up	1.68 (0.68, 4.16)	0.261
Gastritis at baseline	0.8 (0.45, 1.42)	0.44
Gastritis at follow‐up	0.76 (0.45, 1.29)	0.313
Aspirin use	1 (0.63, 1.6)	0.993
PPI use	1.31 (0.91, 1.88)	0.141
Blood group (ref: A)		
B	3.32 (1.1, 10.03)	**0.034**
O	0.96 (0.38, 2.42)	0.934
AB	3.3 (0.4, 27.48)	0.269
Hemoglobin	0.93 (0.8, 1.09)	0.378

CI, confidence interval; GIM, gastric intestinal metaplasia; HR, hazard ratio; PPI, proton pump inhibitor; PUD, peptic ulcer disease.

**Figure 1:**
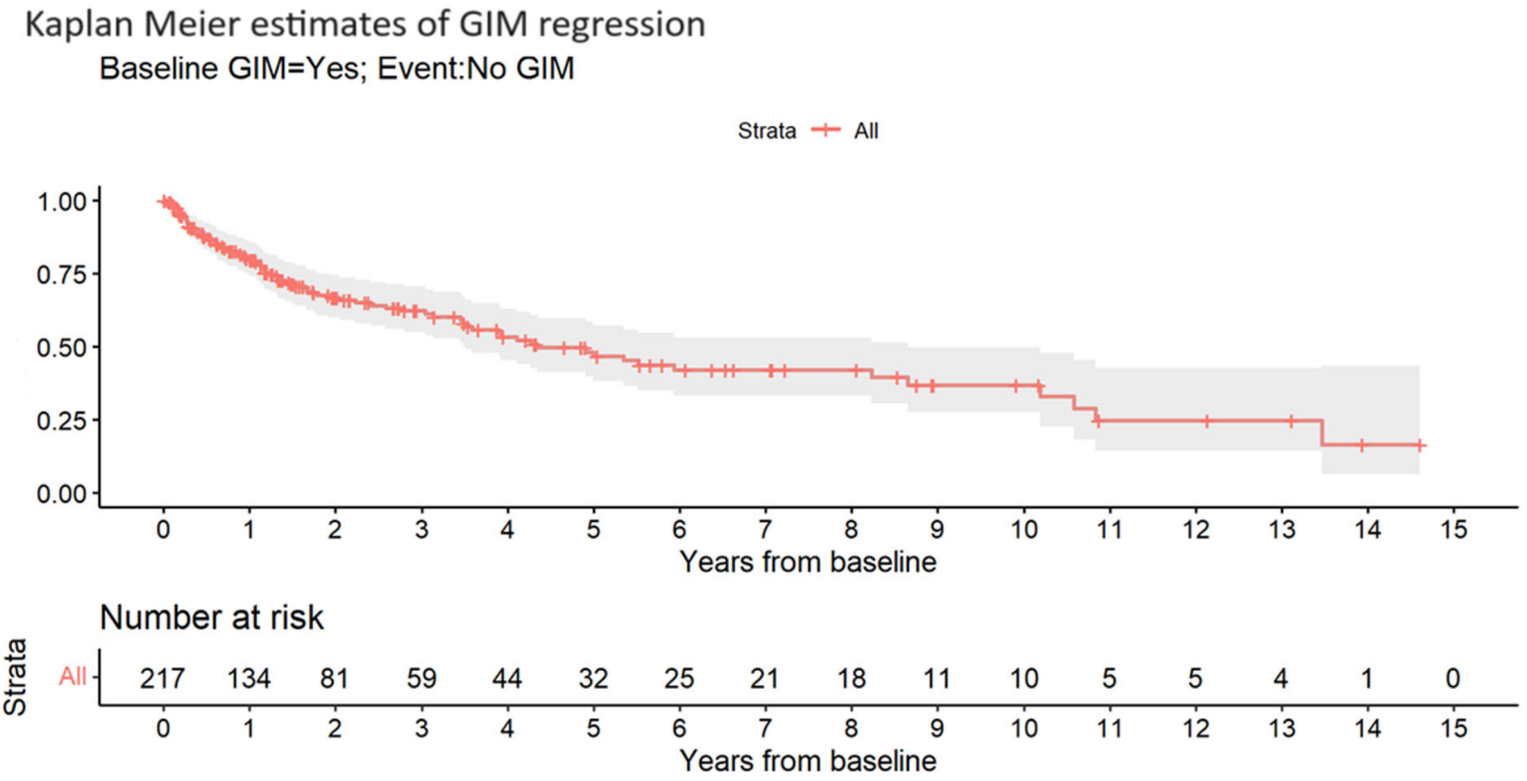
Kaplan–Meier estimates of gastric intestinal metaplasia regression.

In multivariate analysis, the presence of PUD at the time of GIM detection continued to show a higher rate of regression (HR 2.46 [1.24–4.89], *P* = 0.013), as shown in Table 4. The Kaplan–Meier estimate for GIM regression, depicted in Figure 1, indicates that after 5 years of follow‐up, the rate of GIM regression approached 50%.

**Table 4:** Multivariate of factors associated with GIM regression

Predictor	HR (95%CI)	*P* value
*H. pylori* (ref: baseline: no, follow‐up: no)		
Baseline: no, follow‐up: yes	1.18 (0.46, 3.03)	0.731
Baseline: yes, follow‐up: no	0.92 (0.49, 1.7)	0.778
Baseline: yes, follow‐up: yes	0.94 (0.34, 2.6)	0.906
Age at baseline	0.99 (0.98, 1.01)	0.504
Smoking (ref: never)		
Previous	0.54 (0.3, 0.96)	**0.038**
Current	0.62 (0.32, 1.22)	0.169
Peptic ulcer disease at baseline	2.46 (1.24, 4.89)	**0.013**
PPI usage	1.78 (1.11, 2.85)	**0.016**

CI, confidence interval; GIM, gastric intestinal metaplasia; HR, hazard ratio; PPI, proton pump inhibitor; PUD, peptic ulcer disease.

Further subgroup analyses of cases with detailed gastric mapping conducted in 43.3% of patients on follow‐up EGD subsequent to a prior GIM diagnosis also showed that 28.7% had GIM regression, while 71.3% displayed persistent GIM. Subgroup regression analysis showed that African Americans, Hispanics, and individual of other races/ethnicities exhibited lower rates of GIM regression compared with Caucasians, with corresponding HRs of 0.17 (*P* = 0.027), 0.07 (*P* = 0.037), and 0.12 (*P* = 0.042), respectively. PUD at the time of GIM diagnosis was observed in 7.4% of patients in the GIM regression group and 3.0% in the GIM persistence group.

## Discussion

In this retrospective longitudinal investigation, we have evaluated gastric intestinal metaplasia (GIM) regression and the potential factors influencing this phenomenon over time. Notably, within our study population, a substantial 39.1% exhibited GIM regression during the study period. This rate of regression is consistent with findings from multiple studies, where regression of GIM has been observed with or without active intervention.^12^, ^13^ One retrospective study conducted in a Thai population comprising 171 GIM patients reported a regression rate of 57.1% during a follow‐up period spanning 22 months.^6^ Lower rate of GIM regression in our study compared with the study from Thailand is likely due to shorter follow‐up duration for the GIM regression group in our study.

Previous studies have shown an increased age with higher rates of GIM formation, progression, and gastric cancer incidence.^6^, ^13^, ^18^ However, the relationship between age and GIM regression rate has not been extensively explored. Our results showed no age‐related differences between the GIM persistence and regression groups, a trend confirmed by further regression analysis. Similarly, the role of race and ethnicity, recognized as pivotal determinants of GIM status, particularly within the United States population, has been the focus of numerous investigations.^11^, ^19^ Hispanics and African Americans, in particular, have exhibited higher rates of GIM formation and progression when compared with the non‐Hispanic Caucasian population. To our knowledge, our study is the first to investigate the impact of ethnicity/race on GIM regression within the U.S. population. Our subgroup analysis on patients with follow‐up gastric mapping showed that Hispanics and African Americans had lower rates of regression over time compared with Caucasians. Thus, Hispanic and African Americans with higher risks of GIM formation and progression, coupled with reduced GIM regression rates as shown in our study have known higher risks of gastric cancer, warranting a careful consideration of intensified surveillance strategies.

While gastritis is commonly linked to GIM and is known to contribute to GIM's increased risk, our study reveals no significant association between gastritis and the rate of GIM regression. PUD has long been recognized for its relevance in the pathophysiology of gastric cancer. Earlier literature on Japanese patients revealed that individuals with duodenal ulcers had lower rates of gastric cancer, GIM, and atrophic gastritis.^20^, ^21^ Moreover, a clinical trial in China also demonstrated that duodenal ulcers at baseline endoscopy independently reduced the rates of GIM progression. Conversely, the presence of gastric ulcers did not significantly influence GIM progression over a five‐year follow‐up period.^13^ In the context of GIM regression, our study revealed that PUD at the time of GIM diagnosis independently correlated with higher rates of GIM regression. However, it is unclear whether PUD has any direct relationship with GIM or is an indirect marker of underlying pathophysiologic changes of GIM, potentially confounded by therapeutic interventions administered; thus, the causal link between PUD and GIM progression/regression will need further investigations in the future.

Our study found a low rate of *H. pylori* infection among GIM patients, with only 24.9% showing evidence of infection. This is consistent with local rates from previous studies, which ranged from 9.6% to 25.6%.^22^, ^23^ During follow‐up, the persistent infection rate was 13.4%. This contrasts with other studies on *H. pylori* eradication's effect on GIM regression and progression, where baseline infection rates ranged between 70% and 80%.^6^, ^24^ This low rate of *H. pylori* infection in our study groups provides a unique opportunity to examine non‐*H. pylori* factors impacting on GIM regression. Surprisingly, we observed no significant differences in *H. pylori* infection rates between the GIM regression and GIM persistence groups, and the effect of *H. pylori* on GIM regression was not significant. This observation may be attributed to the low rate of *H. pylori* infection at baseline and follow‐up. It is noteworthy that various studies have presented conflicting evidence regarding the correlation between *H. pylori* eradication and GIM regression, with some indicating a positive association,^6^, ^13^, ^24^ while others, including our own, have not substantiated this correlation.^12^, ^25^ While the evidence supporting *H. pylori* eradication's benefits is robust, particularly irrespective of GIM status, its specific impact on GIM remains unclear, especially within regions characterized by low *H. pylori* prevalence.

The role of tobacco use as an important risk factor for gastric cancer has garnered increased attention in recent years, particularly concerning its involvement in the development of gastric premalignant lesions. A substantial cross‐sectional study conducted in Texas, USA, demonstrated that both active and former smokers had a higher risk of GIM when compared with never smokers, with this elevated risk being more pronounced in African Americans, independent of *H. pylori* status.^25^ However, the effect of tobacco use on GIM regression has not been studied extensively. Our study intriguingly reveals a lower rate of regression in current smokers, but not in former smokers, when compared with never smokers. In contrast, a clinical trial conducted in the Thai population did not uncover significant differences in regression rates between smokers and non‐smokers.^6^ These results suggest higher risks of development of GIM in combination with lower rates of GIM regression with smoking thus providing a potential explanation of higher risk of gastric cancer in smokers. Presently, recommendations by the American Gastroenterology Association do not specifically consider smokers in the screening or surveillance of GIM thus gastric cancer screening,^17^ underscoring the need for more comprehensive research to elucidate the effect of smoking on GIM progression, and regression, particularly in the context of gastric cancer risk.

Our study is a first to examine GIM regression within the US population, specifically in an African American and Hispanic American predominant study population. We acknowledge that our retrospective study design may introduce selection bias, potentially underestimating the GIM regression rate, as subjects without subsequent biopsies could have regressed lesions that were not documented. Additionally, we recognize that each sample was assessed by a single pathologist, and the severity and subtype of GIM were not reported. However, the presence of a substantial proportion of high GIM risk non‐Caucasian individuals in our study cohort offers a unique opportunity to dissect the factors influencing GIM regression within these high‐risk populations. Additionally, the low prevalence of *H. pylori* infection among our study participants also provided a unique opportunity to examine non‐*H. pylori* factors affecting GIM regression.
